# *Plasmodium vivax* Infection in Duffy-Negative People in Africa

**DOI:** 10.4269/ajtmh.17-0461

**Published:** 2017-09-07

**Authors:** Peter A. Zimmerman

**Affiliations:** Professor of International Health, Genetics and Biology, The Center for Global Health & Diseases, Case Western Reserve University, Cleveland, Ohio

The understanding that red blood cells (RBCs) lacking the Duffy receptor are resistant to blood stage infection by *Plasmodium vivax* has provided the conceptual path for productive investigation into malaria parasite invasion of the human RBC over the last 40 years.^1^ Following the lead that malaria parasites co-opt RBC surface proteins to provide orientation to, and enable merozoite manipulation of the RBC surface, has made it possible to identify many proteins involved in RBC invasion.^2^

With the availability of the polymerase chain reaction (PCR), increased sensitivity and specificity have significantly improved detection and classification of malaria parasites. Compatibility of PCR assays with microwell formatting has dramatically increased the capacity and processing speed of large patient sample numbers.^3^ As a result of PCR diagnosis, malaria epidemiology studies have easily grown in size from hundreds to ten-thousands of samples. With these diagnostic advances, many new perspectives have been gained in malaria field epidemiology.^4,5^

Most germane to this discussion, an increasing number of studies have identified Duffy-negative people around the world, and specifically across Africa (where this phenotype originated and predominates),^6^ who were PCR-positive for *P. vivax* in Africa and South America (Figure 1 and Supplemental Table 1). In addition, *P. vivax* infection has been detected by either PCR-based or serological methods in African populations in which Duffy-negativity is considered to approach genetic fixation. Among these studies, three manuscripts have recently appeared in the *American Journal of Tropical Medicine and Hygiene* (*AJTMH*).

**Figure 1.**
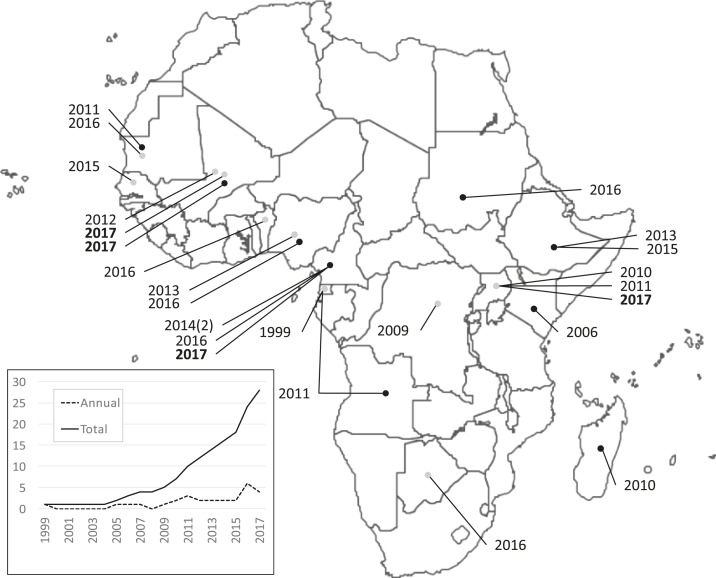
Field-based studies that have reported *Plasmodium vivax* infection in Duffy-negative people across Africa. Dates refer to the year in which manuscripts reporting these findings were published. Black dots represent studies that confirmed Duffy-negative genotype and *P. vivax* infection by PCR methods and gray dots those that detected *P. vivax* infection by either PCR or serological methods. The inset graph tracks the annual (dotted line) and cumulative (solid line) number of manuscripts that have reported *P. vivax*-positive/Duffy-negative malaria.

In this issue of *AJTMH*, Asua et al.^7^ report their observations related to treatment-seeking children from health centers in 10 of Uganda’s 111 districts. From the total of 499 samples, malaria microscopy and rapid diagnostic tests (RDTs) were used to perform preliminary analyses; PCR (18S rRNA) was used to confirm and increase species specificity of their diagnoses. Although the majority of malaria cases involved *Plasmodium falciparum*, 7.8% were nonfalciparum infections and *P. vivax* was found in four children (0.8%).^7^ These authors use their observations to call attention to the importance of species-specific diagnosis to inform adequate antimalarial drug treatment. In particular, because their diagnosis detected *P. vivax* and *P. ovale* infections, treatment to cure patients completely of liver stage hypnozoites would require use of primaquine.^7^ Their paper raises a wider concern that malaria elimination will require the ability to diagnose and develop strategies against all human malaria species being transmitted within endemic regions.

Also, in this issue of *AJTMH*, Niangaly et al.^8^ report their observations from a longitudinal molecular diagnostic survey of *P. vivax* and *P. falciparum* in children under 6 years of age living in Bandiagara, Mali. Their sample collections aligned with rainy and dry season time-points from June 2009 to June 2011. Again, most of the PCR-based diagnostic results (18S rRNA) detected *P. falciparum*. However, 25 children demonstrated *P. vivax*–positivity at single or multiple time-points. *Plasmodium vivax* infections of the Duffy-negative children were submicroscopic infections (PCR-positive/microscopy-negative). Given their observations, the authors stress the importance of molecular diagnostics for understanding *P. vivax* epidemiology in this population. They suggest that their observations of *P. vivax*–positive/Duffy-negative malaria differ from previous reports in which Duffy-positive and Duffy-negative people lived within the same communities because in the new report, *P. vivax* transmission occurred within a fully Duffy-negative resident population. With the possibility that *P. vivax* can be transmitted in the absence of Duffy-positive people, the authors call attention to the new understanding that *P. vivax*–positive/Duffy-negative malaria will complicate African malaria elimination strategies.^8^ Moreover, the authors suggest that *P. vivax* may be gaining efficiency for invading Duffy-negative RBCs, increasing its capacity for stable transmission and causing illness. Speculation suggests that surveys of archived filter paper blood spots or Giemsa-stained blood smears may provide further insight into this claim.

A broader perspective on the prevalence of *P. vivax* in Duffy-negative human populations emerges from serodiagnostic surveillance of antibody responses across Africa. Rogier et al.^9^ reported in the February 2017 issue of *AJTMH* their serodiagnostic results from 805 Malian elementary school children from the regions of Mopti, Sikasso, Koulikoro, and the Bamako capital district. Of these children, 140 (17.4%) carried antibodies against *P. vivax* MSP-1_19._ Similar findings were reported by Poirier et al.^10^ through serodiagnosis of 1,234 blood samples collected from healthy adults (over 18 years old), who visited Departmental Blood Transfusion Centers in Benin for blood donation between 2009 and 2010. In the Beninese study population, 28.7% of patients carried antibodies against rPvMSP1, 21.6% against rPvCSP1, and 15.2% against both. In 84 of these samples selected for additional nested-PCR analyses, 13 were positive for *P. vivax,* and all hosts were genotyped as Duffy-negative.^10^ From serological studies of this nature, it is possible to gain insight into the history and geographical extent to which Duffy-negative populations have been infected with *P. vivax*.

Taken together, available reports continue to document the emerging perspective of wide-spread infection of Duffy-negative populations with *P. vivax*. Going forward, it will be of interest to malaria researchers to continue updating the global map of *P. vivax* to refine what we understand about the geographical range of this parasite,^11^ drug resistance genotypes and phenotypes,^12^ naturally occurring variation in its RBC invasion protein repertoire,^13–17^ and the human and nonhuman primate hosts it infects.^18,19^ Recent studies have added significantly to the map of global *P. vivax* distribution^20^ and provided a focused assessment of this parasite in Africa.^21^ In addition, similar geographical modeling efforts have contributed to our understanding of the relapse characteristics associated with *P. vivax* strains and the influence of temperate versus tropical distribution.^22^ To eliminate *P. vivax,* we will require improved knowledge on all of these fronts to reduce the potential for unknown reservoirs that enable this resilient parasite to continue successful transmission and persist as a cause of significant human disease.

## Supplementary Material

Supplemental Table.
