# Supplementary material for: Plasmodium vivax Infection in Duffy-Negative People in Africa
Source: Am J Trop Med Hyg. 2017 Sep 7;97(3):636–8. doi: 10.4269/ajtmh.17-0461 (PMC5590613; doi:10.4269/ajtmh.17-0461)
Supplement: Supplementary file 1 [file tpmd170461.SD1.pdf]

Supplemental Table 1

Manuscripts reporting evidence of *Plasmodium vivax* infection among Duffy-negative people

PCR detection of *P. vivax* and Duffy genotype					
1	1999	Rubio JM, et al.	Equatorial Guinea	*AJTMH* 60(2): 183–187	PMID:10072133
2	2006	Ryan JR, et al.	Kenya	*AJTMH* 75(4): 575–581	PMID:17038676
3	2010	Menard D, et al.	Madagascar	*PNAS* 107(13): 5967–5971	PMID:20231434
4	2010	Clark TD, et al.	Uganda	*PLoS ONE* 5(7): e11759	PMID: 20689585
5	2011	Dhorda M, et al.	Uganda	*PLoS ONE* 6(5): e19801	PMID:21603649
6	2011	Mendes C, et al.	Angola, Equatorial Guinea	*PLoS NTD* 5(6): e1192	PMID:21713024
7	2011	Wurtz N, et al.	Mauritania	*Malar J* 10: 336	PMID:22050867
8	2013	Peletiri I, et al.	Nigeria	*Int J Malaria Res Rev* 1: 12–21	–
9	2013	Woldearegai TG, et al.	Ethiopia	*Trans R Soc Trop Med Hyg* 107(5): 328–331	PMID:23584375
10	2014	Ngassa Mbenda HG, et al.	Cameroon	*PLoS ONE* 9(8): e103262	PMID:25084090
11	2014	Fru-Cho J, et al.	Cameroon	*Malar J* 13: 170	PMID:24886496
12	2015	Lo E, et al.	Ethiopia	*Malar J* 14: 84	PMID:25884875
13	2016	Abdelraheem MH, et al.	Sudan	*Trans R Soc Trop Med Hyg* 110: 258–260	PMID:27076512
14	2016	Ngassa Mbenda HG, et al.	Cameroon	*J Infect Dev Ctries* 10: 682–686	PMID:27367020
15	2017	Russo G, et al.	Cameroon	*Malar J* 16(1): 74	PMID:28196496
16	2017	Niangaly A, et al.	Mali	*AJTMH* 97(3):743–751	
17	2007	Cavasini CE, et al.	Brazil	*Malar J* 6: 167	PMID:18093292
18	2012	Carvalho TA, et al.	Brazil	*Malar J* 11: 430	PMID:23259672
PCR or serological detection of *P. vivax* and regional inference of Duffy blood group phenotype					
19	2005	Herrera S, et al.	Colombia	*AJTMH* 73(S5): 44–49	PMID:16291766
20	2009	Culleton R, et al.	Congo	*J Infect Dis* 200(9): 1465–1469	PMID:19803728
21	2012	Bernabeu M, et al.	Mali	*Malar J* 11: 405	PMID:23217064
22	2015	Niang M, et al.	Senegal	*Malar J* 14: 281	PMID:26186936
23	2016	Ayorinde AF, et al.	Nigeria	*J Infect Public Health* 9(1): 52–59	PMID:26256113
24	2016	Motshoge T, et al.	Botswana	*BMC Infect Dis* 16(1): 520	PMID:27682611
25	2016	Poirier P, et al.*	Benin	*Malar J* 15(1): 570	PMID:27887647
26	2016	Moukah M, et al.	Mauritania	*Malar J* 15: 204	PMID:27068219
27	2017	Rogier E, et al.	Mali	*AJTMH* 96(2):312–318	PMID:27895279
28	2017	Asua V, et al.	Uganda	*AJTMH* 97(3):752–756	–

* Poirier et al. confirmed *Plasmodium vivax* and Duffy-negative status by polymerase chain reaction methods for 13 individuals.
